# An Approach to the Production of Soluble Protein from a Fungal Gene Encoding an Aggregation-Prone Xylanase in *Escherichia coli*

**DOI:** 10.1371/journal.pone.0018489

**Published:** 2011-04-08

**Authors:** Yilin Le, Jingjing Peng, Huawei Wu, Jianzhong Sun, Weilan Shao

**Affiliations:** 1 Research Center for Biotechnology and Biomass Energy and College of Life Sciences, Nanjing Normal University, Nanjing, Jiangsu, PR China; 2 Biofuels Institute, School of Environment, Jiangsu University, Zhenjiang, Jiangsu, PR China; University of Kent, United Kingdom

## Abstract

The development of new procedures and protocols that allow researchers to obtain recombinant proteins is of fundamental importance in the biotechnology field. A strategy was explored to overcome inclusion-body formation observed when expressing an aggregation-prone fungal xylanase in *Escherichia coli*. pHsh is an expression plasmid that uses a synthetic heat-shock (Hsh) promoter, in which gene expression is regulated by an alternative sigma factor (σ^32^). A derivative of pHsh was constructed by fusing a signal peptide to *xynA2* gene to facilitate export of the recombinant protein to the periplasm. The xylanase was produced in a soluble form. Three factors were essential to achieving such soluble expression of the xylanase: 1) the target gene was under the control of the Hsh promoter, 2) the gene product was exported into the periplasm, and 3) gene expression was induced by a temperature upshift. For the first time we report the expression of periplasmic proteins under the control of an Hsh promoter regulated by σ^32^. One unique feature of this approach was that over 200 copies of the Hsh promoter in an *E. coli* cell significantly increased the concentration of σ^32^. The growth inhibition of the recombinant cells corresponded to an increase in the levels of soluble periplasmic protein. Therefore, an alternative protocol was designed to induce gene expression from pHsh-ex to obtain high levels of active soluble enzymes.

## Introduction

*Escherichia coli* is extensively used for industrial production of recombinant proteins due to its well-characterized genetics and ability to grow rapidly to a high density on inexpensive substrates [1]. However, it is a common problem for recombinant protein aggregates to form inclusion bodies in the cytoplasm and/or periplasm upon gene over-expression in *E. coli* [2], [3]. This complex phenomenon has many contributing factors: insolubility of the product at the concentrations being produced, inability to fold correctly in the bacterial environment, or lack of appropriate bacterial chaperone proteins [4], [5]. Several strategies are commonly employed to reduce inclusion bodies: a) controlling the rate of protein synthesis, b) enabling secretion into the periplasm, and c) co-expression of chaperone genes. The control of the rate of protein synthesis can be achieved by changing the promoter to regulate the level of expression, fusing the target gene to another gene, and adjusting the growth conditions, such as pH and temperature of the medium [6]. The secretion of recombinant proteins into the periplasm of *E. coli* has also been shown to benefit the production of certain recombinant proteins resulting in higher solubility of the gene product, correct folding, and facilitated downstream processing [1], [5], [7], [8], [9], [10], although inclusion bodies may still form in the periplasm [11], [12]. The *E. coli* periplasm is an oxidizing environment that contains a series of chaperones or enzymes promoting the appropriate folding of proteins [13], [14], [15], [16]. Some *E. coli* periplasmic proteins such as DegP and FkpA participate in the folding of certain secreted recombinant proteins [17], [18], [19]. Finally, increasing the concentration of chaperones in a heat-shock system regulated by σ^32^ in *E. coli* has been shown to assist in the correct folding of the target protein [20], [21], [22], and co-expression of DnaK-DnaJ can greatly increase the soluble proportion of recombinant proteins in the cytoplasm [22].

The gene *xynA* of *Thermomyces lanuginosus*, a thermophilic fungus, encodes a thermostable GF11 endoxylanase. This xylanase is free of cellulase activity, and hydrolyses xylan to produce xylose and xylooligosaccharides [23]. Recently, the DNA sequence of *xynA* has been optimized, and intracellular expression of the enzyme in *E. coli* has reached a high level [24]. However, the recombinant enzyme XynA was mainly found in inclusion bodies, and only a small proportion was soluble and active [24]. We tried to express the xylanase at lower temperature using pET expression system [25], but the solubility of XynA was not significantly improved.

Protein aggregation is the major bottleneck for the production of recombinant protein in microbial organisms and it is known that there are no universal approaches to overcome this problem. Heat-shock proteins have been shown to assist in the correct folding of the target protein [22]. pHsh is an expression plasmid that uses a synthetic heat-shock (Hsh) promoter to control target gene, in which gene expression is regulated by an alternative σ^32^ [26]. In order to explore an approach to overcoming inclusion-body formation, we report on the construction of a new vector pHsh-ex, which enables the export of target proteins into the periplasm of *E. coli* cells, and its unique properties in the expression of soluble xylanase from *xynA*. This paper also reports on the effect of the high level expression of periplasmic proteins on cell growth, and the effect of pHsh plasmids on σ^32^ levels in recombinant cells.

## Results

In a previous study, the xylanase gene *xynA* from *T. lanuginosus* was mutated to *xynA2* for an increase of intracellular expression in *E. coli*. Because the mutation was performed without change of amino acid sequence, *xynA2* encodes the same XynA as that encoded by the wild-type gene. The over-expression of this fungal enzyme resulted in the production of large amount of inclusion bodies with a weak xylanase activity of about 47 U/ml [24]. To reduce inclusion body formation of XynA, we expressed the gene by using different strategies. These included a) inducing simultaneous expression of *xynA2* and chaperone genes by using pHsh vector, b) producing periplasmic protein in vector pET-20b(+), c) fusing a xylosidase/arabinosidase gene into the 5′ end of *xynA2*, and d) expressing the xylanase gene in a new vector pHsh-ex, which combined the functions of heat-shock (Hsh) promoter and protein secretion.

**Figure 1:**
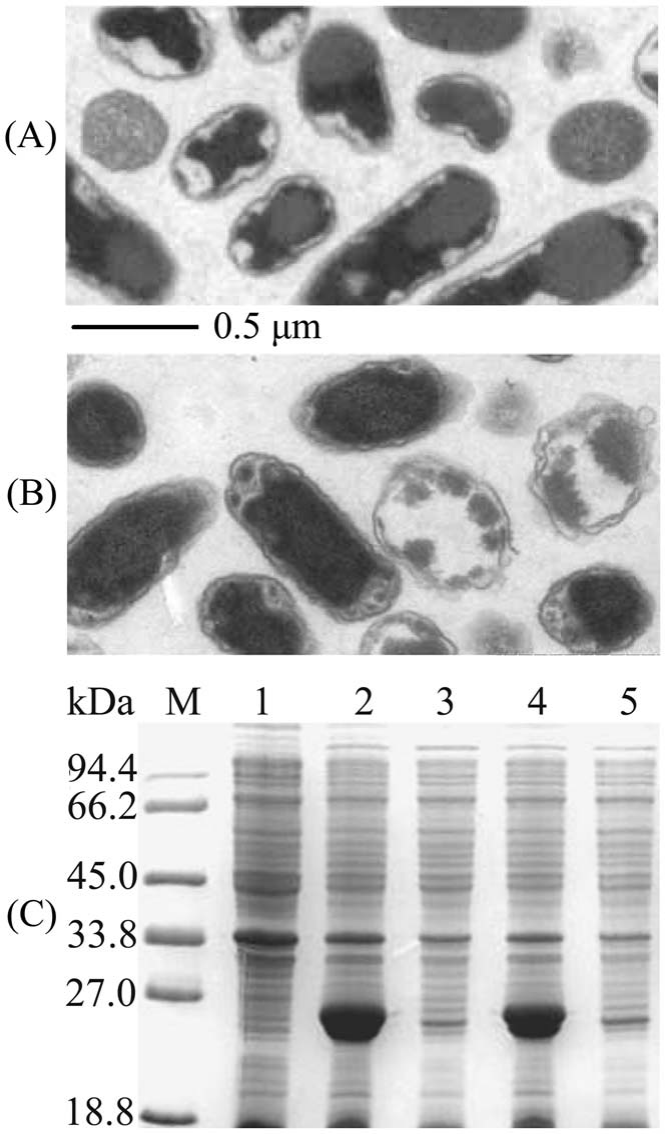
Inclusion bodies formation of XynA expressed from plasmids pHsh-*xynA*2 and pET-*xynA*2. **A–B**. Electronic microscopy observation of inclusion bodies in the pHsh-*xynA*2 transformed *E. coli* cells (A) and pET-*xynA*2 transformed *E. coli* cells (B), ultrathin section. **C**. SDS-PAGE analysis of XynA expressed from pET-*xynA*2. Lanes: M, protein marker; 1, total protein of *E. coli* containing pET-20b(+) without target gene; 2, total protein and 3, soluble protein of *E. coli* containing pET-*xynA*2 grown at 37°C; 4, total protein and 5, soluble protein of *E. coli* containing pET-*xynA*2 grown at 20°C.

Expression vector pHsh has a copy number of over 200, and controls the expression of a target gene via σ^32^ because it comprises a synthetic Hsh promoter and the replication origin from pUC18/19 [26]. A temperature upshift can cause a rapid increase of σ^32^ that immediately activates the transcription of the target gene in pHsh as well as the heat-shock proteins including a group of chaperones of *E. coli*. However, the simultaneous expression of heat-shock chaperones did not reduce the inclusion body formation of intracellular expressed XynA in *E. coli* cells harboring plasmid pHsh-*xynA2* (Fig. 1A). Therefore, various expression plasmids were further constructed (Table 1) for following efforts to reduce inclusion body formation from XynA.

**Table 1:** Plasmids.

Plasmid	Relevant characterization	Source or reference
pHsh	Expression vector, heat-shock (Hsh) promoter, ori of pUC18/19, Amp^r^	GenBank accession no: FJ571619
pET-20b(+)	Expression vector,T7 promoter, *pelB* leader	Novagen
pHsh-ex	Expression vector, Hsh promoter, *ompA* leader	This study
pHsh-*xynA*2	Expression of *xynA2*, Hsh promoter, intracellular protein	Yin et al. (24)
pHsh-*xar-xyn*	Expression of fused *xarA*/*xynA2*, Hsh promoter, intracellular protein	This study
pET-*xynA*2	Expression of *xynA2*, T7 promoter, periplasmic protein	This study
pHsh-ex*-xynA*2	Expression of *xynA2*, Hsh promoter, periplasmic protein	This study
pHsh-ex-*xar-xyn*	Expression of fused *xarA*/*xynA2*, Hsh promoter, periplasmic protein	This study
pHsh-ex-*xynA-*m1	Expression of *xynA-m1*, Hsh promoter, periplasmic protein	This study
pHsh-*xynB*	Expression of *xynB*, Hsh promoter, intracellular protein	Wu et al. (29)
pLac-*xynB*	Expression of *xynB*, *lac* promoter, intracellular protein	This study

### Expression of periplasmic protein from pET-20b(+)

The gene *xynA2* was cloned and fused with the signal peptide sequence in pET-20b(+) to generate plasmid pET-*xynA*2. A high level expression was achieved by the pET-*xynA*2 transformed *E. coli* cells grown at either 37°C or 20°C; however, only a small proportion of XynA was soluble in the cell-free extracts, and most of the enzyme was in inclusion bodies (Fig. 1B, C). SDS-PAGE analysis revealed that XynA expressed from pET-*xynA2* was partitioned into two adjacent bands, suggesting that some of the enzyme molecules were still carrying uncleaved signal peptide (Fig. 1C). These results indicate that the export of XynA into periplasm by using pET-*xynA*2 did not significantly enhance the soluble protein yield.

Using the same plasmid, we tried to control the rate of protein synthesis by cultivating the recombinant cells at the temperature as low as 20°C. When grown at 37°C and 20°C, the pET-*xynA*2 transformed cells produced 24±3 and 36±2 U/ml of xylanase, respectively. The enzyme activity increased by about 52% when the cell growth was slowed by cultivation at 20°C, compensating for the effect of the rate of gene expression rate on the formation of inclusion bodies, although the overall proportion of soluble recombinant enzyme was not significantly improved in a gel when similar volumes of the solution were loaded for SDS-PAGE (Fig. 1C).

The possible effect of a heat-shock induction for the expression of chaperones on the inclusion body formation was also examined by using the same plasmid. We performed a temperature shift from 30°C to 42°C over the pET-*xynA*2 transformed cells during IPTG induction. However, this heat-shock did not increase the soluble yield of recombinant protein, but rather decreased the growth of the cells by about 12% (data not shown).

### Construction and expression of fused genes

**Figure 2:**
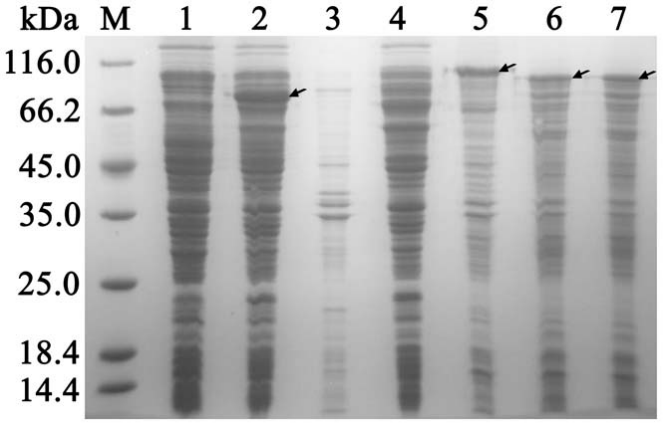
SDS-PAGE analysis of inclusion bodies produced from the fused genes. Lanes: M, protein markers; 1, whole cell protein of pHsh transformed *E. coli*; 2–3, expression of XarB from pHsh-*xarB* transformed *E. coli*, showing soluble protein (lane 2) and insoluble protein (lane 3) fractions; 4–5, expression of fused gene of *xarB* and *xynA2* in plasmid pHsh-*xar-xyn*, showing soluble protein (lane 4) and insoluble protein (lane 5) of *E. coli*; 6, inclusion bodies of fused XarB and the C-terminus of XynA N-terminus of XynA; 7, inclusion bodies of fused XarB and the C-terminus of XynA. Arrows indicate the bands o f recombinant proteins.

As commonly recognized, fusing the target gene to another gene could reduce inclusion bodies. The gene *xarB* from *Thermoanaerobacter ethanolicus* encodes a thermostable bifunctional xylosidase/arabinosidase with two 85 kD subunits [27], [28]. After *xarB* was subcloned into pHsh, the overexpression was achieved in pHsh-*xarB* transformed *E. coli* to produce XarB in soluble form (Fig. 2). We fused *xarB* to the 5′ end of *xynA*2 in pHsh-*xynA2* to examine whether a multifunctional xylan degrading enzyme could be produced in soluble form. Unfortunately, when an intracellular protein was expressed from the fused gene *xar-xyn* in pHsh-*xar-xyn*, XarB failed to rescue XynA from the inclusion body formation, but was captured into precipitant by XynA (Fig. 2). To determine the motifs that caused inclusion body formation, we truncated *xynA2* to the fragments encoding only 100 amino acids of N-terminus or C-terminus, and fused each fragment to the 3′ end of *xarB*. SDS-PAGE analysis showed that the fusion of either N-terminal or C-terminal polypeptide to the C-terminus of XarB could lead to inclusion body formation although a subunit of XarB was composed of 784 amino acids while a XynA fragment was only 100 amino acids (Fig. 2).

### Soluble periplasmic protein produced from *xynA2* in the pHsh-ex

In an effort to reduce the formation of inclusion bodies, we added a DNA sequence coding for signal peptide into the cloning sites of pHsh to obtain a new vector pHsh-ex. The target protein fused with the signal peptide in pHsh-ex could be exported into the periplasm. For the first time we are reporting here the expression of periplasmic proteins under the control of an Hsh promoter regulated by σ^32^.

**Figure 3:**
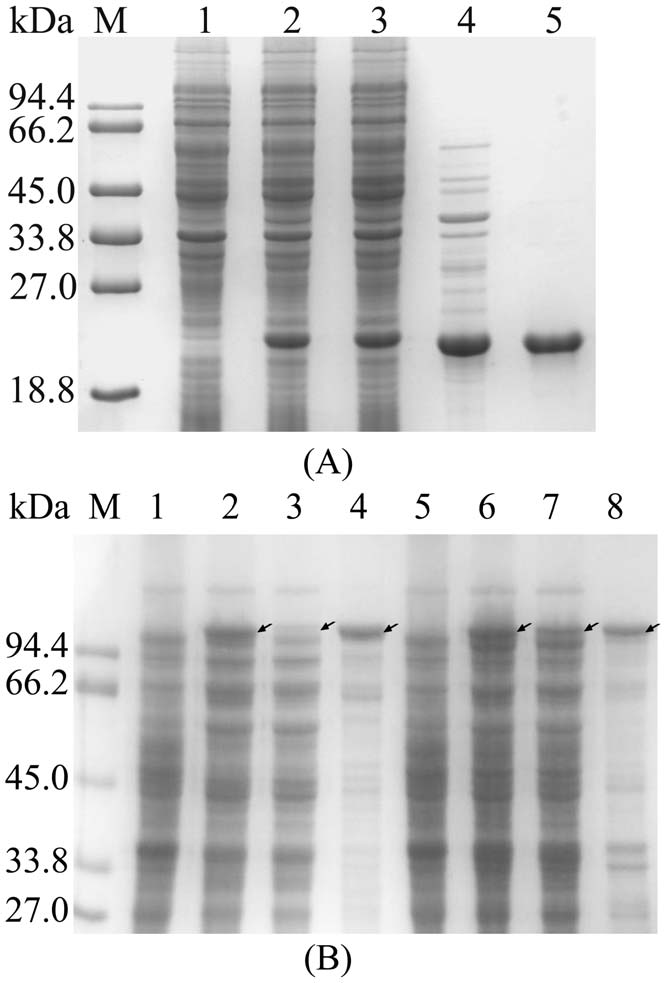
SDS-PAGE analysis for the expression of recombinant proteins in pHsh-ex. **A**. Soluble expression of XynA from pHsh-ex-*xynA2*; lanes: M, protein markers; 1, total protein of *E. coli* containing pHsh-ex; 2, total protein and 3, soluble protein of *E. coli* containing pHsh-ex-*xynA*2; 4, periplasmic protein obtained by cold osmotic shock; 5, XynA purified by ion exchange chromatography. **B**. Comparison of fused protein expressed from plasmids pHsh-*xar-xyn* and pHsh-ex-*xar-xyn*; lanes: M, protein markers; 1, total protein of *E. coli* containing pHsh; 2–4, total protein, soluble protein and insoluble protein of *E. coli* containing pHsh-*xar-xyn*, respectively; 5, total protein of *E. coli* containing pHsh-ex; 6–8, total protein, soluble protein and insoluble protein of *E. coli* containing pHsh-ex-*xar-xyn*, respectively. Arrows indicate the bands of recombinant proteins.

Interestingly, when *xynA*2 was expressed by using vector pHsh-ex, the xylanase migrated in a single band in SDS gels, and there was no detectable decrease of protein from this band after insoluble components were removed from cell lysates by centrifugation (Fig. 3A). The xylanase activity produced by cells harboring pHsh-ex-*xynA*2 reached 221±5 U/ml (319±7 U/mg protein). The xylanase was purified in three steps: cold osmotic shock, heat treatment and DEAE-Sepharose FF column (Table 2); after heat treatment followed by chromatography on DEAE-Sepharose FF, protein in the xylanase active peak showed a single band on SDS-PAGE gel with a molecular mass of 22.5 kDa (Fig. 3A). In comparison with the expression of periplasmic protein from pET-*xynA2*, only in vector pHsh-ex the inclusion body formation from *xynA*2 was primarily avoided by exporting the recombinant protein into the periplasm (Fig. 1C and Fig. 3A). The findings listed in Table 3 indicate that three factors were essential to achieving the soluble expression of XynA: 1) the target gene was under the control of the Hsh promoter, 2) the gene product was exported into the periplasm, and 3) gene expression was induced by a temperature upshift.

**Table 2:** Purification of recombinant xylanase from *E. coli* harboring pHsh-ex-*xynA*2.

Steps	Total protein(mg)	Total activity(U)	Specific activity (U/mg)
Periplasmic fraction	29.4	20609	701
Heat treatment	21.2	17214	812
DEAE Sepharose FF	3.1	4644	1498

**Table 3:** Conditions to produce soluble protein from xynA2.

Plasmid	Hsh promoter	Signal peptide	Temperature upshift	Inclusion-body formation
pHsh-*xynA*2	+^a^	−a	+	+++^a^
pET-*xynA*2	−	+	+	+++
pHsh-ex-*xynA*2	+	+	+	ND^b^

a : +, Yes; −, no; +++, majority.

b : ND, not detectible.

The fused gene *xar-xyn* was also cloned into pHsh-ex to generate pHsh-ex*-xar-xyn*. SDS-PAGE analysis revealed that the level of soluble Xar-Xyn protein of 108 kD was significantly increased by using pHsh-ex to export the recombinant protein to periplasm (Fig. 3B). However, the major proportion of the recombinant protein remained insoluble probably because with a molecular mass of 108 kD, the recombinant protein was too big to be exported into periplasm.

### Enhanced protein expression from mutant *xynA-m1*

Previously, intracellular expression of xylanase was improved by sequence optimization by site-directed mutagenesis without changing the protein sequence [24]. To obtain a pHsh-ex plasmid with increased protein expression, we constructed a mutant library by error-prone PCR, and screened the transformants on the LB plates supplemented with 2% xylan which produced transparent zones of different sizes around their colonies. Approximately 3,000 recombinant bacteria were screened, and only the mutant with a relative large transparent zone was used for further analysis. From these mutants, a plasmid was isolated and designated as pHsh-ex-*xynA-*m1 (Table 1). The mutated xylanase gene, *xynA-*m1, was sequenced, and a single mutation point was found at codon 180, where a GGT was mutated to AGT (G180S).

In comparison, the recombinant cells produced higher xylanase activity from *xynA-m1* than from *xynA2*; the former was 247±3 U/ml culture (409±3 U/mg total cell protein) while the latter was 221±5 U/ml (319±7 U/mg). After the enzymes were purified, the specific activities of XynA from *xynA2* and XynA^m1^ from *xynA-m1* were 1498±13 and 1509±15 U/mg (a variation of about 0.75%), respectively. There were no significant differences observed in enzyme activity and stability between the wild-type and mutant enzymes in our extensive characterizations.

### Effects of expression level of soluble periplasmic protein on cell growth

**Figure 4:**
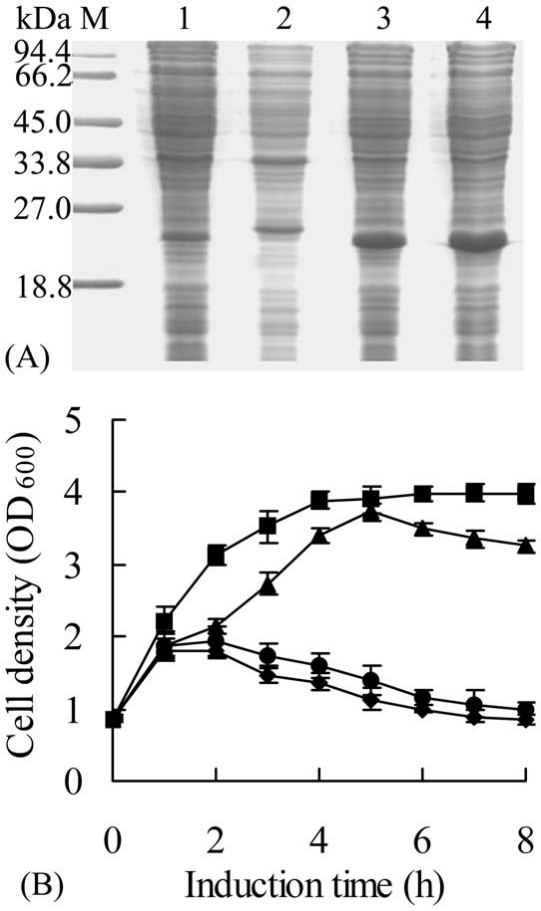
The expression of soluble xylanase and cultivation profile of *E. coli* harboring pHsh-*xynA*2, pET-*xynA*2, pHsh-ex-*xynA*2 and pHsh-ex-*xynA*–m1. **A**. SDS-PAGE analysis of the soluble xylanase; Lanes: M, protein marker; 1–4, soluble protein of *E. coli* containing pHsh-*xynA*2, pET-*xynA*2, pHsh-ex-*xynA*2, and pHsh-ex-*xynA*-m1, respectively. **B**. Cultivation profiles of recombinant *E. coli* cells after induction. Symbols: cells harbored plasmid pHsh-*xynA*2 (-▪-), pET-*xynA*2 (-▴-), pHsh-ex-*xynA*2 (-•-) or pHsh-ex-*xynA*-m1 (-♦-).

Compared with pHsh-*xynA*2 which produces intracellular recombinant protein, pET-*xynA*2 (Fig. 1), pHsh-ex*-xynA*2 or pHsh-ex-*xynA*-m1 gave recombinant cells a different growth profile during heterologous expression (Fig. 4). Meanwhile, when the protein from about equal numbers of cells were loaded in each lane, SDS-PAGE showed that *E. coli* cells harboring pHsh-ex*-xynA*-m1 produced higher levels of soluble recombinant xylanase than those harboring pHsh-ex-*xynA*2 and pET-*xynA*2 (Fig. 1 and Fig. 4A). High level expression of soluble periplasmic protein was associated with poor cell growth during heterologous expression of the xylanase gene using the plasmids (Fig. 4B). Following standard procedures [24], [26], the cells harboring pHsh-*xynA2* grew for up to 6 h to a cell density of OD_600_ 3.9 after heat-shock induction. The cells harboring pET-*xynA2* also grew to a maximal cell density of about OD_600_ 3.5 when *E. coli* BL 21(DE3) was used as host to express periplasmic xylanase with large proportion in inclusion bodies (Fig. 4B). However, the cells harboring pHsh-ex-*xynA2* stopped growing within 2 h after heat-shock induction, which resulted in the low production of total biomass as well as recombinant protein. A further decrease of growth was observed with the cells transformed by pHsh-ex-*xynA*-m1, which produced 28% more active xylanase than pHsh-ex-*xynA2* (Fig. 4). These results imply that the expression of soluble secreted protein from pHsh-ex-*xynA2* or pHsh-ex-*xynA*-m1 exceeded the tolerable levels for *E. coli* and resulted in a growth inhibition.

**Figure 5:**
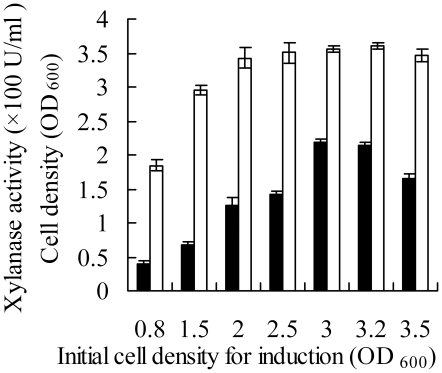
The effect of initial cell density for induction on the production of recombinant periplasmic protein in the *E. coli* cells harboring pHsh-ex-*xynA*2. Symbols: -□- cell density; -▪- xylanase activity.

When pHsh vectors were used to express intracellular proteins, the optimal induction time was determined to be around a cell density of OD_600_ 0.8. In order to compensate for the short growth phase of over-expressed periplasmic protein from pHsh-ex, an optimal alternative protocol was established on the basis of the induction time, final cell densities and xylanase activities. Figure 5 shows that the highest cell density could be reached when the temperature-shift was performed to induce expression at an initial cell density OD_600_ 2.0, 2.5, 3.0 or 3.2, but the best enzyme activity could be obtained by performing the temperature shift at about OD_600_ 3.0.

### The pHsh vector in *E. coli* cells causes significant variations in the concentration of σ^32^

The findings have indicated that only protein export resulting from the gene expression in pHsh-ex, and heat-shock of cells carrying that vector could uniquely increase soluble protein expression in *E. coli* cells (Table 3). To examine the effect of the pHsh vector on the physiology of *E. coli* without interferences of inclusion body formation, T7 RNA polymerase expression, or overloading the cell membrane, we employed pHsh-*xynB* to express an intracellular soluble xylanase. A control plasmid, pLac-*xynB*, was constructed from pHsh-*xynB* by replacing the Hsh promoter with the *lac* promoter so that excluded all other possible differences when the intracellular protein from the recombinant cells harboring these plasmids were analyzed by Western blotting.

**Figure 6:**
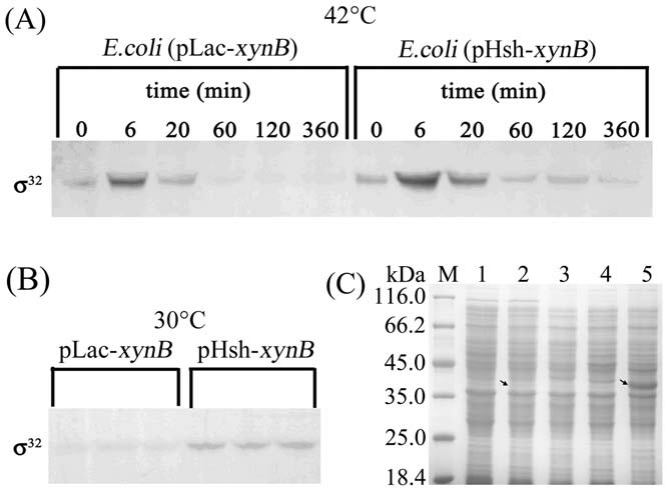
Immuno-detection of σ^32^ in *E. coli* K-12 cells carrying Hsh vector, and over-expression of XynB in pHsh. **A**. The variation of σ^32^ concentrations in cells subjected to heat-shock induction. Recombinant cells carrying pLac-*xynB* or pHsh-*xynB* were grown to OD_600_ = 0.7 at 30°C then transferred into a 42°C water-bath incubator; samples were withdrawn at various timepoints and cell densities were determined; cells harvested from samples were re-suspended in the volume of 1.5×SDS sample buffer adjusted to a equivalent cell density of OD_600_ = 30, and 10 µl of lysates were loaded for immuno-blotting. **B**. Detection of σ^32^ in 6 transformants grown at 30°C; *E. coli* cells were transformed by pLac-*xynB* or pHsh-*xynB*, single colonies were grown to OD_600_ = 3 (about 6 h), and cells were harvested from 1 ml culture and re-suspended in 0.1 ml of lysis buffer (1.5×SDS), and 15 µl samples were loaded on SDS-gel for immuno-blotting after incubated in boiling water bath for 10 min. **C**. Expression of XynB from pLac-*xynB* and pHsh-*xynB*, Lanes: M, protein markers; 1–3, gene expression tests for pLac-xynB, showing intracellular protein of recombinant cells without induction, and with IPTG induction and heat-shock induction, respectively; 4 and 5, intracellular protein of pHsh and pHsh-*xynB* transformed cells induced by heat-shock, respectively. Arrows indicate the bands of recombinant proteins.

The results indicated that the σ^32^ protein accumulated to the highest level within about 6 min, and returned to normal levels within about 1 hour after a temperature shift was performed on the cells harboring pLac-*xynB* as well as pHsh-*xynB* (Fig. 6A). Here we found that the σ^32^ levels in the recombinant cells were significantly different between the cells harboring pHsh-*xynB* and pLac-*xynB*. Under either a constant temperature of 30°C or after a temperature shift from 30°C to 42°C, the σ^32^ levels were much higher in the cells harboring pHsh than in those transformed with non-pHsh plasmid, pLac (Fig. 6). This result explains the observation that the expression of the genes in pHsh vectors, e.g. pHsh-*xynB* (Fig. 6C), can last for up to 6 hours to reach high levels, while in natural *E. coli* cells, heat-shock protein synthesis rates normally peak at about 5 min after a temperature upshift and then rapidly decline to a steady-state level at ambient temperature [29].

## Discussion

Inclusion bodies are densely packed particles of aggregated protein found in the cytoplasmic or periplasmic spaces of *E. coli* during high-level expression of heterologous protein [2], [4]. But practically, most people would not examine whether there are dense electron-refractive particles, and simply believe that all recombinant proteins in precipitants are in inclusion bodies, which can include unstable proteins denatured during cell growth or after cell disruption. Fungal enzyme XynA is thermostable with a relatively small molecular mass; it forms typical inclusion bodies in both the cytoplasmic and periplasmic spaces of *E. coli* during high-level expression under normal conditions (Fig. 1A, B).

In some previous studies, the improvement in soluble protein levels was attributed to the fact that the periplasmic space provides a more oxidative environment than the cytoplasm [7], [30], [31]. However, the oxidative environment did not seem to benefit the soluble expression of XynA because the insoluble xylanase was also observed from pET-*xynA*2 as a mature protein, which should exist in the periplasm (Fig. 1B, C). Co-expression of chaperones such as DnaK-DnaJ can greatly increase the soluble yields of aggregation-prone proteins [21]. Heat-shocking the cells to induce expression of a foreign gene can also activate the synthesis of chaperones [32]. In recent years, several periplasmic proteins including DegP and FkpA were identified as chaperones [17], [18]. DegP and FkpA play a role in the folding of certain periplasmic proteins [3], [19]. Furthermore, DegP and FkpA can be induced to express by temperature upshift via the periplasm specific σ^E^ heat-shock regulon [33]. In this work, the mature protein expressed from the gene in pET-*xynA*2 formed inclusion bodies, and a temperature upshift upon the recombinant cells could not improve the soluble yield (data not shown).

Interestingly, increased soluble expression was achieved from the recombinant genes in pHsh-ex-*xynA*2 or pHsh-ex-*xynA*-m1; the cells harboring pHsh-ex-*xynA*2 produced soluble XynA, which gave an activity level 6–9 times higher than that expressed from pET-*xynA*2. Compared with the expression from pHsh-*xynA*2 and pET-*xynA*2, we found that the increase in the soluble expression of the xylanase gene from pHsh-ex-*xynA*2 or pHsh-ex-*xynA*-m1 depends upon 3 factors: 1) the target gene is under the control of the Hsh promoter, 2) the gene product is exported into the periplasm, and 3) the expression is induced by a temperature upshift (Table 3). The dependence on the Hsh promoter for increased expression is the most unique of these factors, although the others are also essential. These results imply that not the periplasm specific σ^E^ heat-shock regulon but σ^32^ heat-shock regulon contributes to the soluble expression of XynA and XynA^m1^.

The dependence of the Hsh promoter reflects a unique physiological process in the *E. coli* cells carrying pHsh plasmids. Skelly *et al.* reported the correlation between the σ^32^ levels and *in vitro* expression of *E. coli* heat-shock genes [34]. When the heat-shock transcription factor σ^32^ was overexpressed in *E. coli*, enzyme activity of preS2-S9-b-galactosidase was increased [21]. An increased concentration of σ^32^ was observed in the cells carrying an expression plasmid with >200 copies of Hsh promoter. The findings not only explain how high level expression is achieved in this expression system, but also indicate that σ^32^ of *E. coli* could play an important role in the soluble expression of XynA from pHsh-ex.

While the mechanism for the pHsh-ex mediated soluble expression of an aggregation-prone protein is not completely understood, this paper could offer a strategy to prevent or reduce the inclusion body formation of some proteins such as vaccine antigens, antibodies, therapeutic proteins, and enzymes provided that the gene expression level is high enough. By using the pHsh-ex vector for expression of periplasmic proteins, the recombinant cells stopped growth within about 2–3 h after a temperature upshift (Fig. 3B). This phenomenon could have resulted from overload of the cell membrane, which is supported by the fact that the mutation from *xynA*2 to *xynA*–m1 caused an increase of gene expression along with a significant decrease of the final cell density. Therefore, alternative induction protocols can be designed to fulfill the necessity of a large number of starting cells. The culture and induction techniques used in this work should be applicable to the performance of pHsh-ex mediated expression of other proteins to be exported into the periplasm.

In summary, it is extremely important and useful to develop new techniques and protocols to solve inclusion body formation and inactivation of soluble recombinant proteins. The soluble expression of an aggregation-prone xylanase in *E. coli* was achieved by using pHsh-ex as expression vector. An alternative protocol was designed to induce gene expression from pHsh-ex to overcome the growth inhibition corresponded with the increase of soluble periplasmic protein expression. The dependence of the Hsh promoter reflects a unique physiological process in the *E. coli* cells carrying pHsh plasmids which caused a significant increase of σ^32^ in the host cells.

## Materials and Methods

### Bacterial strains, plasmids and growth media

The *E. coli* JM109 (Promega) and BL21(DE3) (Novagen) strains were used as hosts for gene cloning and protein expression, respectively. *E. coli* cells were grown in Luria-Bertani (LB) medium supplemented with ampicillin (100 µg ml^−1^), and the cell growth was monitored by measuring the optical density at 600 nm (OD_600_). For comparison, a commercially available T7 system expression vector, pET-20b(+) (Novagen) was used to evaluate the secretive expression of *xynA*2. The expression plasmid pHsh-amp (GenBank accession no: FJ571619) was used to construct a new vector for the expression of periplasmic protein. Plasmids pHsh-*xynA*2 [24] and pHsh-*xynB* [26] were used to determine cell growth profiles and the σ^32^ levels in the recombinant cells.

### DNA manipulation

DNA isolation, amplification, digestion and ligation were performed by following standard procedures [35] and manufacturers' instructions. Plasmid DNA and PCR products were purified using the Qiagen Plasmid kit and PCR purification kit (Qiagen, USA). DNA restriction and modification enzymes were purchased from TaKaRa (PR China). DNA transformation was performed by electroporation using GenePulser (Bio-Rad, USA).

### Construction of expression plasmids

The nucleotide sequence coding for the signal peptide of the OmpA protein (GenBank accession no. NC_000913) was synthesized in the primers ex-N and ex-C (Table 4), and introduced into pHsh by an inverse PCR using the high-fidelity *Pyrobest* DNA polymerase (TaKaRa); PCR product was phosphorylated and self-ligated to generate new vector pHsh-ex (GenBank accession number of FJ715939).

**Table 4:** Primers.

*Primers*	Sequence (Bases at restriction sites are in bold case)
ex-N	5′-AGTGCAACTGCAATCGCGATAGCAGTCTTTTTCATGGGTATATCTCCTTC-3′
ex-C	5′-TGCTGGTTTCGCTACCGTAGCGCAGGCTGCTCCGAAAGAGGCCTCTAGACTGCAG-3′
X1	5′-CTCAGACCACCCCGAACTCTGAAGGCTG-3′
X2	5′-CCC**AAGCTT**T- TAGCCAACGTCAGCAACAG-3′
X3	5′-CCC**GATATC**ATGCAGACTACCCC- GA-3′
X4	5′-CCG**CTCGAG**GCCAACGTCAGCAACA-3′
Lac1	5′-GGAAACATTATGTTGATCCAATGACCTGTTA-3′
Lac2	5′-CCCACATGTAAACTCTTCCGCTTCCTCT-3′
Lac3	5′-GATA- ACAATTTCACACAAGGAGATATACCCATGG-3′
Lac4	5′-CGCTCACAATTCCACACAACATAATGTTTCCCCC-3′
xarB-N	5′-GCAAGCCATTATATTTAGATTC-3′
xarB- C	5′-CCC**CTCGAG**CTATTTATTCTCTACCCTTAC-3′
P1	5′-AAGCCATTATATTTAGATTCC-3′
P2	5′-TTTATTCTCTACCCTTACTTC-3′
P3	5′-CAGACTACCCCGAACAG-3′
P4	5′-CATATGTTTTTCTCCTTCTTG-3′
N1	5′-TAAAAGCTTGAAGGCCGCTTC -3′
N2	5′-AGACGGGTCGTAAGTACCGA-3′
C1	5′-TCTGGTGCTACTGACCTGGG-3′
ex-x-x-C	5′-CCCCTCGAGTTAGCCAACGTCAGCAACAG-3′

The xylanase gene was amplified from the plasmid pHsh-*xynA2* [24] with primers X1 and X2, or X3 and X4 (Table 4), and cloned into pHsh-ex or pET-20b(+) at the respective *Stu* I/*Hind* III sites or *EcoR* V/*Xho* I sites. Therefore, the xylanase gene was fused with the *ompA* leader sequence in pHsh-ex and *pelB* leader in pET-20b(+), respectively, to generate pHsh-ex-*xynA2* and pET-*xynA2* (Table 1).

The control plasmid pLac-*xynB* was constructed by replacing the Hsh promoter in pHsh-*xynB* with the *lac* promoter, which was performed through 2 rounds of inverse PCR using the following two pairs of primers: Lac1 and Lac2, Lac3 and Lac4 (Table 4).

The *xarB* gene from *T. ethanolicus* was amplified from the genome with primers xarB-N and xarB-C (Table 4). PCR products were purified, digested with corresponding restriction enzyme(s), and ligated to pHsh at the respective *Stu* I/*Xho* I sites, to generate pHsh-*xarB*.

The primers P1 and P2 were used to amplify *xarB* gene from the plasmid pHsh-*xarB* while P3 and P4 were employed to amplify linear pHsh-*xynA2* by using *Pyrobest* DNA polymerase. Then, *xarB* was ligated to linear pHsh-*xynA2* to generate vector pHsh-*xar-xyn*. The fused gene *xar-xyn* was also sub-cloned into pHsh-ex with primers ex-x-x-C and xarB-N to generate pHsh-ex*-xar-xyn*. The fragments were amplified from the plasmid pHsh-*xar-xyn* with primers N1, N2 or C1, P4, respectively, to generate pHsh-*xar-xyn-N100* or pHsh-*xar-xyn-C100*.

### Construction and screening of a mutant library

A mutant library was constructed by using error-prone PCR on *xynA2* with pHsh-ex-*xynA2* as template. A 20 µl of reaction mixture contained 0.2 µg primers, 2.0 mM MgCl_2_, 0.2 mM dNTPs each, 0.25 mM MnCl_2_, 1.25 units (U) of *Taq* DNA polymerase (TaKaRa). DNA amplification was carried out in 15 cycles of 94°C, 1 min, 72°C, 1.5 min, and 60°C, 2 min, and a final incubation at 60°C for 10 min. The mutants were expressed in pHsh-ex in *E. coli*, and screened on the plates containing 2% xylan and corresponding antibiotics.

### Expression and purification of recombinant xylanase

The *E. coli* BL 21 (DE3) cells harboring pET-*xynA2* were grown at 20°C or 37°C, and gene expression was induced by addition of IPTG (isopropyl-β-D-thio galactopyranoside) to a final concentration of 1 mM. The cells transformed with pHsh-*xynA*2, pHsh-ex-*xynA*2, pHsh-*xar-xyn*, pHsh-ex-*xynA*-m1, pHsh-ex-*xar-xyn*, pHsh-*xynB*, pHsh-*xar-xyn-N100* or pHsh-*xar-xyn-C100* were grown at 30°C, and induced to express xylanase by transferring the test tubes or flasks into a water-bath shaking incubator, which had been pre-heated to 42°C.

The recombinant enzyme was isolated from the periplasm by cold osmotic shock according to a published protocol [31]. The cells were harvested by centrifugation (6,000×g for 5 min) from a 1 L culture were re-suspended in 100 mM Tris-HCl containing 20% sucrose and 1 mM EDTA (pH 8.0), and then pelleted by centrifugation (8,000×g for 5 min) followed by re-suspension in ice-cold water for 10 min. After the addition of MgCl_2_ to a final concentration of 1 mM, the cell suspension was incubated on ice for a further 10 min before being pelleted by centrifugation (8,000×g for 5 min). The supernatant was mixed with 100 mM Tris-HCl buffer (pH 8.0) in a ratio of 1∶1, and incubated in a 65°C water bath for 20 min followed by centrifugation (20,000×g, 30 min, 4°C). The resulting supernatant was loaded onto a DEAE Sepharose FF (Amersham) column (2.5×15 cm), which had been pre-equilibrated with 50 mM Tris-HCl buffer (pH 8.0). Proteins bound to the column were eluted with a linear gradient of 0–0.2 mM NaCl in the same buffer.

### Enzyme activity assay

Xylanase activity was determined by the 4-hydroxybenzoic acid hydrazide method [36] with oat spelt xylan (Sigma, USA) as substrate. The reaction mixture comprised of 100 µl 0.5% (w/v) oat spelt xylan in water, 90 µl phosphate buffer (50 mM, pH 6.0) and 10 µl properly diluted enzyme. The reaction was conducted at 65°C for 10 min, and stopped by adding 600 µl of 4-hydroxybenzoic acid hydrazide solution into the reaction mixture. The reducing sugar was determined by reading the absorbance at 410 nm after the test tubes were incubated for 10 min in a boiling water bath and then cooled on ice. One unit of xylanase activity was defined as the amount of enzyme releasing 1 µmol reducing sugar per min.

### Transmission Electron Microscopy

Recombinant *E. coli* was washed three times with 0.1 M sodium cacodylate buffer (pH 7.4), fixed cells in the same buffer containing 2.5% glutaraldehyde at 4°C for 2 hrs and embedded in epoxy resin. Ultra-thin sections were double-stained in uranyl acetate and lead citrate.

### Protein analysis and Western blotting

Protein concentrations were determined by using the Bio-Rad Protein Assay Kit II based on the Bradford dye-binding method. The whole-cell protein and the soluble protein in cell-free extracts were prepared by disrupting cells suspended in 50 mM Tris-HCl buffer (pH 8.0) by sonication, followed by centrifugation at 12,000×g for 10 min. Recombinant proteins were observed on an SDS-PAGE (15%) gel stained with Coomassie blue R-250. The protein bands in the gel were analyzed by density scanning with an image analysis system (Bio-Rad).

The σ^32^ proteins in *E. coli* K-12 transformed by pHsh-*xynB* or pLac-*xynB* were detected by immuno-blotting. The transformants were cultivated at 30°C with or without the up-shift of temperature to 42°C, and samples were withdrawn at various points over a timecourse and immediately frozen with −70°C ethanol. After thawing on ice, the cells were centrifuged at 4°C and re-suspended in a volume of SDS-PAGE sample buffer normalized according to the approximate cell density at time of the harvest and lysed by heating at 100°C for 5 min. The proteins in the cell lysates were separated on a 12% SDS-PAGE gel, and transferred electrophoretically to a 0.45 µm polyvinylidene difluoride membrane (Biotrace PVDF, Millipore) at 250 mA at 4°C for 1.5 h. The membrane was incubated with a blocking solution consisting of 5% nonfat dry milk in TBST (150 mM NaCl, 50 mM Tris, 0.1% Tween-20, pH 7.5) at room temperature (RT) for 1 h. After washing 4 times (10 min each) with TBST, the blocked membrane was incubated in a sealed bag with 1∶1000 diluted monoclonal antibody to σ^32^ (Neoclone) at 4°C overnight. The membrane was rinsed 3 times for 5 min with TBST, and incubated with 1∶3000 diluted horseradish peroxidase (HRP)-conjugated goat anti-rabbit IgG antibody (γ-chain specific) at RT for 1.5 h. The membrane was washed 4 times for 5 min with TBST, and then incubated with TMB (3, 3′, 5, 5′-tetramethylbenzidene) stabilized substrate for HRP (Promega) to develop the blue color according to the manufacturer's instructions.

## References

[1] Baneyx F (1999). Recombinant protein expression in *Escherichia
coli*.. Curr Opin Biotechnol.

[2] Bowden GA, Paredes AM, Georgiou G (1991). Structure and morphology of protein inclusion bodies in
*Escherichia coli*.. Bio/Technology.

[3] Pan KL, Hsiao HC, Weng CL, Wu MS, Chou CP (2003). Roles of DegP in prevention of protein misfolding in the
periplasm upon overexpression of penicillin acylase in *Escherichia
coli*.. J Bacteriol.

[4] Singh SM, Panda AK (2005). Solubilization and refolding of bacterial inclusion body
proteins.. J Biosci Bioeng.

[5] Sevastsyanovich YR, Alfasi SN, Cole JA (2010). Sense and nonsense from a systems biology approach to microbial
recombinant protein production.. Biotechnol Appl Biochem.

[6] Strandberg L, Enfors SO (1991). Factors influencing inclusion body formation in the production of
a fused protein in *Escherichia coli*.. Appl Environ Microbiol.

[7] Choi JH, Lee SY (2004). Secretory and extracellular production of recombinant proteins
using *Escherichia coli*.. Appl Microbiol Biotechnol.

[8] De Maio A (1999). Heat shock proteins: facts, thoughts, and dreams.. Shock.

[9] Mergulhão FJM, Summers DK, Monteiro GA (2005). Recombinant protein secretion in *Escherichia
coli*.. Biotechnol Adv.

[10] Missiakas D, Raina S (1997). Protein misfolding in the cell envelope of *Escherichia
coli*: new signaling pathways.. Trends Biochem Sci.

[11] Bowden GA, Paredes AM, Georgiou G (1991). Structure and morphology of protein inclusion bodies in
*Escherichia coli*.. Bio/Technology.

[12] Hunke S, Betton JM (2003). Temperature effect on inclusion body formation and stress
response in the periplasm of *Escherichia
coli*.. Mol Microbiol.

[13] Berndt C, Lillig CH, Holmgren A (2008). Thioredoxins and glutaredoxins as facilitators of protein
folding.. Biochim Biophys Acta.

[14] Chen J, Song JL, Zhang S, Wang Y, Cui DF (1999). Chaperone activity of DsbC.. J Biol Chem.

[15] Miot M, Betton JM (2004). Protein quality control in the bacterial
periplasm.. Microb Cell Fact.

[16] Shao F, Bader MW, Jakob U, Bardwell JC (2000). DsbG, a protein disulfide isomerase with chaperone
activity.. J Biol Chem.

[17] Arie JP, Sassoon N, Betton JM (2001). Chaperone function of FkpA, a heat shock prolyl isomerase, in the
periplasm of *Escherichia coli*.. Mol Microbiol.

[18] Kadokura H, Kawasaki H, Yoda K, Yamasaki M, Kitamoto K (2001). Efficient export of alkaline phosphatase overexpressed from a
multicopy plasmid requires degP, a gene encoding a periplasmic protease of
*Escherichia coli*.. J Gen Appl Microbiol.

[19] Zhang Z, Song LP, Fang M, Wang F, He D (2003). Production of soluble and functional engineered antibodies in
*Escherichia coli* improved by FkpA.. BioTechniques.

[20] Baneyx F, Mujacic M (2004). Recombinant protein folding and misfolding in *Escherichia
coli*.. Nat Biotechnol.

[21] Thomas JG, Baneyx F (1996). Protein folding in the cytoplasm of *Escherichia
coli*: requirements for the DnaK-DnaJ-GrpE and GroEL-GroES
molecular chaperone machines.. Mol Microbiol.

[22] Thomas JG, Baneyx F (1996). Protein misfolding and inclusion body formation in recombinant
*Escherichia coli* cells overexpressing heat-shock
proteins.. J Biol Chem.

[23] Gomes J, Gomes I, Kreiner W, Esterbauer H, Sinner M (1993). Production of high level of cellulose-free and thermostable
xylanase by a wild strain of *Thermomyces lanuginosus* using
beechwood xylan.. J Biotechnol.

[24] Yin E, Le Y, Pei J, Shao W, Yang Q (2008). High-level expression of the xylanase from *Thermomyces
lanuginosus* in *Escherichia
coli*.. World J Microbiol Biotechnol.

[25] Studier FW, Moffatt BA (1986). Use of bacteriophage T7 RNA polymerase to direct selective
high-level expression of cloned genes.. J Mol Biol.

[26] Wu H, Pei J, Jiang Y, Song X, Shao W (2010). pHsh vectors, a novel expression system of *Escherichia
coli* for the large-scale production of recombinant
enzymes.. Biotechnol Lett.

[27] Shao W, Wiegel J (1992). Purification and characterization of a thermostable
beta-xylosidase from Thermoanaerobacter ethanolicus.. J Bacteriol.

[28] Mai V, Wiegel J, Lorenz WW (2000). Cloning, sequencing, and characterization of the bifunctional
xylosidase-arabinosidase from the anaerobic thermophile thermoanaerobacter
ethanolicus.. Gene.

[29] Wu H, Pei J, Wu G, Shao W (2008). Overexpression of GH10 endoxylanase XynB from *T.
maritima* in *E. coli* by a novel vector with
potential for industrial application.. Enzyme Microb Technol.

[30] Tilly K, Spence J, Georgopoulos C (1989). Modulation of stability of the *Escherichia coli*
heat shock regulatory factor sigma.. J Bacteriol.

[31] Thorstenson YR, Zhang Y, Olson PS, Mascarenhas D (1997). Leaderless polypeptides efficiently extracted from whole cells by
osmotic shock.. J Bacteriol.

[32] de Marco A (2009). Strategies for successful recombinant expression of disulfide
bond-dependent proteins in *Escherichia
coli*.. Microb Cell Fact.

[33] Raivio TL, Silhavy TJ (1999). The sigma E and Cpx regulatory pathways: overlapping but distinct
envelope stress responses.. Curr Opin Microbiol.

[34] Skelly S, Coleman T, Fu C, Brot N, Weissbach H (1987). Correlation between the 32-kDa a factor levels and in vitro
expression of *Escherichia coil* heat shock
genes.. Proc Natl Acad Sci.

[35] Sambrook J, Russell DW (2001).

[36] Lever M (1972). A new reaction for colorimetric determination of
carbohydrates.. Anal Biochem.

